# Multi-Organ Failure in a Patient With Diabetes due to COVID-19 With Clear Lungs

**DOI:** 10.7759/cureus.8147

**Published:** 2020-05-15

**Authors:** Sohaip Kabashneh, Hammad Ali, Samer Alkassis

**Affiliations:** 1 Internal Medicine, Wayne State University - Detroit Medical Center, Detroit, USA

**Keywords:** coronavirus disease (covid-19), multiorgan failure, severe diabetic ketoacidosis, metabolic encephalopathy

## Abstract

The pandemic of coronavirus disease 2019 has emerged in late 2019 infecting millions of people worldwide. Diabetes mellitus (DM) has been associated with severe illness and mortality mainly due to acute respiratory distress syndrome. We report a case of a middle-aged man with DM and COVID-19 who developed seizure and altered mental status, found to have diabetic ketoacidosis (DKA), acute kidney injury, hypovolemic shock, and hyperammonemia all contributing to metabolic encephalopathy. He was admitted to the ICU and subsequently intubated for airway protection; with appropriate management his condition improved and was successfully extubated. The patient had no lung involvement throughout the illness. We report this case to highlight that COVID-19 can lead to multi-organ failure in patients with DM even in the absence of lung involvement which all physicians should be mindful of.

## Introduction

The pandemic of coronavirus disease 2019 (COVID-19), a disease caused by severe acute respiratory syndrome-coronavirus-2 (SARS-CoV-2), has emerged as an easily transmissible disease affecting millions of people worldwide. The spectrum of COVID-19 ranges from mild to critical illness; the majority (80%) of cases are mild and self-limiting; severe disease requiring hospitalization occurs in the remaining 20% mainly due to fulminant pneumonia and respiratory failure [1]. Diabetes mellitus (DM) has been associated with severe illness and mortality [1-3]. The association between DM and COVID-19 in the literature so far has always been in the setting of fulminant pneumonia and respiratory failure. However, COVID-19 is an infection that can certainly trigger diabetic ketoacidosis (DKA) and possibly multi-organ failure even in the absence of lung involvement.

## Case presentation

We report a case of a 54-year-old man with a past medical history of hypertension and DM, who presented to the ED after he had witnessed a new onset generalized tonic clonic seizure at home and then again en route to the hospital. Few days prior, he had decreased oral intake and has not been acting like himself as per his wife; she did not notice vomiting, diarrhea, abdominal pain, fever, cough, or headache. On arrival to the ED he was unresponsive and unable to protect his airway; heart rate was 119 beats/min, blood pressure 100/70 mmHg, and his oxygen saturation was 96% on nonrebreather mask. On exam he was unresponsive, not moving any of his extremities spontaneously; his pupils were equal, round, and reactive to light, he did not have a cough or gag reflex, and no facial droop was noted. His chest and abdomen exam were unremarkable.

The patient was subsequently intubated for airway protection; basic labs showed multiple abnormalities including: DKA with blood glucose (BG) 1100, anion gap 46, HCO_3_ 4 , beta hydroxybutyrate 65.6; he also had acute kidney injury (AKI) with creatinine (Cr) of 4.9 (baseline was 1.0), blood urea nitrogen 84 , potassium 6.4, sodium 146, chloride 96, phosphorus 18.7, lactic acid 17.3; there was also evidence of liver function abnormalities with elevated ammonia level at 244; arterial blood gas was consistent with high anion gap metabolic acidosis with pH 6.79, HCO_3_ 4, PaCO_2_ 36, PaO_2_ 473. Complete blood count revealed elevated white blood cells at 15.8, absolute lymphocyte count 8, hemoglobin 15.2, and platelet 178; urine drug screen was negative.

**Figure 1:**
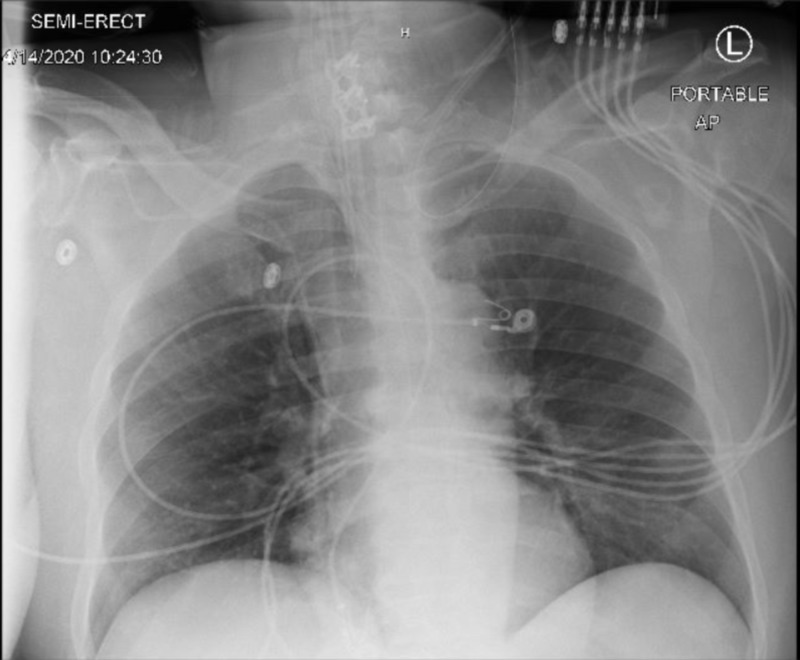
Chest X-ray is unremarkable for infiltrates.

CT head showed no evidence of acute intracranial process, and chest X- ray was unremarkable (Figure 1). Infectious workup was done including COVID-19, urine analysis, urine culture, blood culture, cerebrospinal fluid (CSF) analysis, and CSF culture.

The patient was started on an insulin drip and intravenous fluids for DKA; he was started on levetiracetam to manage the seizures, and broad spectrum antibiotics with cefepime, vancomycin, ampicillin, and acyclovir empirically for meningitis. Electroencephalography (EEG) which was done showed moderate to severe degree of cerebral dysfunction consistent with metabolic/hypoxic encephalopathy, the waveforms were not epileptiform in nature.

On the second day of admission, the patient was still unresponsive. However, DKA has resolved with BG 190, anion gap 10, HCO_3_ 24, and therefore, the insulin drip was discontinued and was started on glargine and correction scale. His AKI started to improve as Cr trended down to 1.5 and potassium 4.5. COVID test came back positive and therefore he was started on hydroxychloroquine; the other infectious workup including urine analysis, urine culture, blood culture, CSF analysis, and CSF culture came back negative. The patient was breathing on minimal ventilator settings but was not extubated because of the mental status.

On the third day, the patient’s mental status improved; he became more awake, alert, and was able to follow simple command and was extubated successfully. Antibiotics were discontinued, repeat labs showed normalization of kidney function, lactic acid trended down to 1.2, ammonia level also improved to 65. Given the overall clinical improvement the patient was transferred to medical floor and discharged after a total of seven days of admission without any evidence of lung involvement.

## Discussion

COVID-19 in severe cases can lead to death, mainly due to acute respiratory distress syndrome, and subsequent acute hypoxic respiratory failure [1-3]. Multiple studies have been conducted to explain the tendency of SARS-CoV-2 to affect the lungs concluding that angiotensin converting enzyme 2 (ACE2), an enzyme abundantly found in the lungs, serves as a portal of entry into cells for the SARS-CoV family [4-5].

Diabetes has been associated with worse outcome in COVID-19 patients, as it has been postulated to enhance viral entry into cells and dampen the host immune response in fighting the infection [2]. Interestingly ACE2 is present in the pancreas mainly the endocrine tissues, i.e. pancreatic islets that regulates blood glucose levels [4]. This finding would explain the involvement of the pancreas and the resulting hyperglycemia seen in patients suffering from SARS-CoV-2, like our patient above. An acute insult on the islet cells would result in hyperglycemia in patients with previously normal islet function and worsening hyperglycemia and potentially triggering DKA in those with a history of DM [6].

Many viruses have been associated with the development of type I DM in humans. SARS-CoV in 2003 was associated with acute hyperglycemia and higher death rates in patients with DM. However, a three-year follow up did not show increased incidence of diabetes [6]. Follow up studies will be needed after the present pandemic to ascertain if same holds true for SARS-CoV-2.

In diabetic patients COVID-19 can lead to DKA and potentially multiorgan failure even without lung involvement like our patient above; thus diabetic patients with COVID-19 require close monitoring of all organs not just the lungs, as those patients seem to be more susceptible to renal failure, encephalopathy, heart involvement, and shock compared to nondiabetics [5].

## Conclusions

Diabetes mellitus has been associated with worse outcome in COVID-19 mainly due to fulminant pneumonia. However, hyperglycemia and DKA can result from COVID-19 leading to altered mental status and admission to ICUs even in the absence of lung involvement. Thus physicians should be mindful of glucose levels in patients admitted for COVID-19 even if they do not have a previous history of diabetes. Physicians should also closely monitor all organs in diabetic patients with COVID-19 instead of just the lungs.
